# *In vivo* tumor imaging of pre-clinical models via reflection-mode measurements of circular degree of polarization

**DOI:** 10.1117/1.JBO.30.S3.S34105

**Published:** 2025-09-05

**Authors:** Michael D. Singh, Héctor A. Contreras-Sánchez, Alex Vitkin

**Affiliations:** a University of Toronto, Department of Medical Biophysics, Temerty Faculty of Medicine, Toronto, Ontario, Canada; b University of Toronto, Department of Radiation Oncology, Temerty Faculty of Medicine, Toronto, Ontario, Canada; c University Health Network, Princess Margaret Cancer Centre, Toronto, Ontario, Canada

**Keywords:** depolarization, tumor, cancer, detection, polarimetry, biophotonics

## Abstract

**Significance:**

Tumor tissues exhibit contrast with healthy tissue in circular degree of polarization (DOP) images via higher magnitude circular DOP values and increased helicity-flipping. This phenomenon may enable polarimetric tumor detection and surgical/procedural guidance applications.

**Aim:**

Depolarization metrics have been shown to exhibit differential responses to healthy and cancer tissue, whereby tumor tissues tend to induce less depolarization; however, the understanding of this depolarization-based contrast remains limited. Therefore, we investigate depolarization signals from tumor tissue and non-tumor tissue.

**Approach:**

Mice (n=3) with human pancreatic ductal adenocarcinoma (PDAC) xenografts enable polarimetric comparison between tumor tissue and non-tumor tissues. Modified signed-value DOP equations aid in the interpretation of DOP images, which encode helicity-flipping and co-linearity as negative values, but still yield the same magnitudes as conventional DOP calculations.

**Results:**

Linear DOP is greater in magnitude than circular DOP across both tissue types; however, circular DOP yields greater contrast between tumor and non-tumor tissues. Circular DOP values are higher in magnitude and more negative (i.e., more helicity-flipping) in tumors, whereas linear DOP values exhibit similar behavior; however, they are only slightly higher in magnitude and slightly more negative (i.e., more co-linearity) in tumors.

**Conclusions:**

Circular DOP images yield useful contrast between human PDAC xenografts and surrounding healthy skin in live mice. Each tumor region exhibited higher magnitude circular DOP (and total DOP) values, as previously observed. We noted three indications of Rayleigh scattering in the tumor tissue: (1) linear DOP > circular DOP, (2) helicity-flipping > helicity-preservation, and (3) co-linear intensity > cross-linear intensity. Rayleigh scatterers have been found to be highly polarization preserving; thus, we posit that higher DOP in tumor tissues may arise from an increased presence of Rayleigh scatterers. Furthermore, circular DOP may yield greater contrast between tumor and non-tumor via its well-observed sensitivity to scatterer size. Further investigation is warranted to test these hypotheses.

## 1 Introduction

One of the major goals in cancer polarimetry is to detect tumor regions in tissue using polarized light to enable surgical or procedural guidance during tumor resections or biopsy sampling.^1^ Some of the key advantages of using polarized light to achieve these goals are non-contact and label-free cancer-to-healthy tissue contrast,^2^ relatively cost-effective polarimetry apparatus,^2^ and the combination of polarization optics with other clinical optical systems [e.g., optical coherence tomography (OCT),^3^ endoscopy,^4^ and mass spectrometry^5^^,^^6^]. To this end, we investigate the polarimetric contrast between tumor and non-tumor tissues in live mice.

Depolarization metrics have yielded contrast between malignant and non-malignant tissues in a growing number of tissue types, including skin, cervix, colon, larynx, oral mucosa, and others.^4^^,^^7^^–^^14^ In reflection-mode polarimetry studies—the necessary geometry for *in vivo* applications—depolarization is typically weaker in malignant tissues than in healthy tissues.^4^^,^^7^^–^^11^^,^^13^^–^^15^ The underlying reasons for this are not yet well understood. Absorption due to increased vascularization is thought to be a factor because absorption shortens photon path lengths and decreases depolarization;^16^^,^^17^ however, weaker depolarization is also found in tumors where absorption differences are absent,^4^^,^^8^^,^^18^ as we also note in this study. Another hypothesis that has been repeatedly cited (see Refs. 19–24) is the “reduction in scattering due to the destruction of normal tissue architectures” leading to weaker depolarization.^4^ However, no direct evidence is provided in Refs. 19–24 to show that scattering diminishes in tumor tissue. In fact, to the contrary, Ref. 20 states that light scattering is enhanced: “we are naturally led to the conclusion that the cancerous tissue with high cellular density and vascularization typical of this region depolarizes less than the other tissues, a characteristic which is certainly connected with the enhancement of light scattering due to cell nuclei and blood vessels.” Indeed, through other more established techniques such as diffuse reflectance^25^ and OCT,^26^ it has been observed that scattering increases, rather than decreases, in tumors, as one might expect if tissue architectures are broken down and heterogeneity increases. Therefore, the common assertion that tumors exhibit higher polarization preservation (i.e., lower depolarization) due to absorption and reduced scattering requires reconsideration, and overall, it appears that our understanding of depolarization contrast between tumor and normal tissue remains limited, particularly as it pertains to scattering mechanisms.

To gain insight into the differential depolarization behavior in tumor and non-tumor tissues, we analyze linear and circular depolarization images of tumors (heterotopic pancreatic adenocarcinoma xenografts) which are surrounded by non-tumor tissue (healthy mouse skin) in live mice. Interestingly, some reflection-mode polarimetry studies have revealed that tumors exhibit depolarization signatures that are associated with small Rayleigh regime scatterers (i.e., scatterers with diameters much smaller than the illumination wavelength). These depolarization signatures were first observed by MacKintosh et al.^27^ who measured the degree of polarization (DOP) of backscattered light from polystyrene microsphere suspensions comprising either small Rayleigh regime spheres (∼0.09  μm diameter) or larger spheres (∼0.6  μm); DOP is calculated as DOP = 1 depolarization, which quantifies the fraction of light that is not depolarized. It was found that suspensions of small Rayleigh regime spheres exhibited (1) higher linear DOP than circular DOP, (2) more helicity flipping than helicity preservation for incident circularly polarized light, and (3) higher co-linear intensity than cross-linear intensity for incident linearly polarized light. Follow-up studies, both experimental and numerical, have mostly reaffirmed these findings.^28^^–^^39^ For details on these findings and underlying mechanisms, we refer the reader to our companion paper.^40^ Rayleigh-associated depolarization signatures (1), (2), and (3) have been observed in tumor tissues via reflection mode polarimetry [see Refs. 10, 14, 19, and 41–44 which correspond to (1), (2), and (3), respectively]. These observations suggest that tumor tissues may comprise higher proportions of small Rayleigh regime scatterers. Indeed, scatterer size has long been thought to play a role in depolarization in tissues.^15^^,^^18^^,^^19^^,^^24^^,^^45^^–^^47^

In addition to the Rayleigh-indicative depolarization signatures noted above, other techniques have suggested a decrease in scatterer size in malignant tissues, for example, Arifler et al.^48^ used a finite-difference time-domain method to show that the scattering cross-sections decrease in neoplastic fibre networks compared with normal networks. Using polarized angular dependent light scattering, Ramachandran et al.^44^ noted that tumorigenic models contain relatively more of the smallest scatterers compared with non-tumorigenic models. Mourant et al.^49^ compared the scatterer size of proliferative versus non-proliferative tumor cells and found that “the average size of the scatterers is smaller for the exponential phase cells” (i.e., proliferative cells). In Ref. 40, it is shown that Rayleigh scatterers yield higher total DOP magnitudes than larger scatterers (scatterers with diameters similar to the illumination wavelength). The evidence presented herein suggests that Rayleigh scattering is enhanced in tumor tissue, which may be a contributing factor to the higher polarization preservation of tumor tissue.

## 2 Methods

### 2.1 Experimental Polarimetric Imaging System

A schematic and detailed description of the experimental setup can be found in Fig. 2 of Ref. 6. Briefly, a 180-deg reflection mode geometry was implemented whereby the detection axis coincided with the illumination axis; this was achieved using a beam splitter. The illumination source was a helium–neon laser at λ=632.8  nm, and the detection device was an intensified-CCD camera (PI-MAX^®^ 3, Princeton Instruments, Acton, Massachusetts, United States). A ∼flat-field beam was used to illuminate each entire specimen. The polarization state generator/analyzer consisted of manually rotated quarter-wave retarders and linear polarizers. The Mueller matrix of the beam splitter was measured and accounted for in the calculation of each polarimetric image (see Ref. 6 for details).

### 2.2 Mouse and Window Chamber Model

**Fig. 1:**
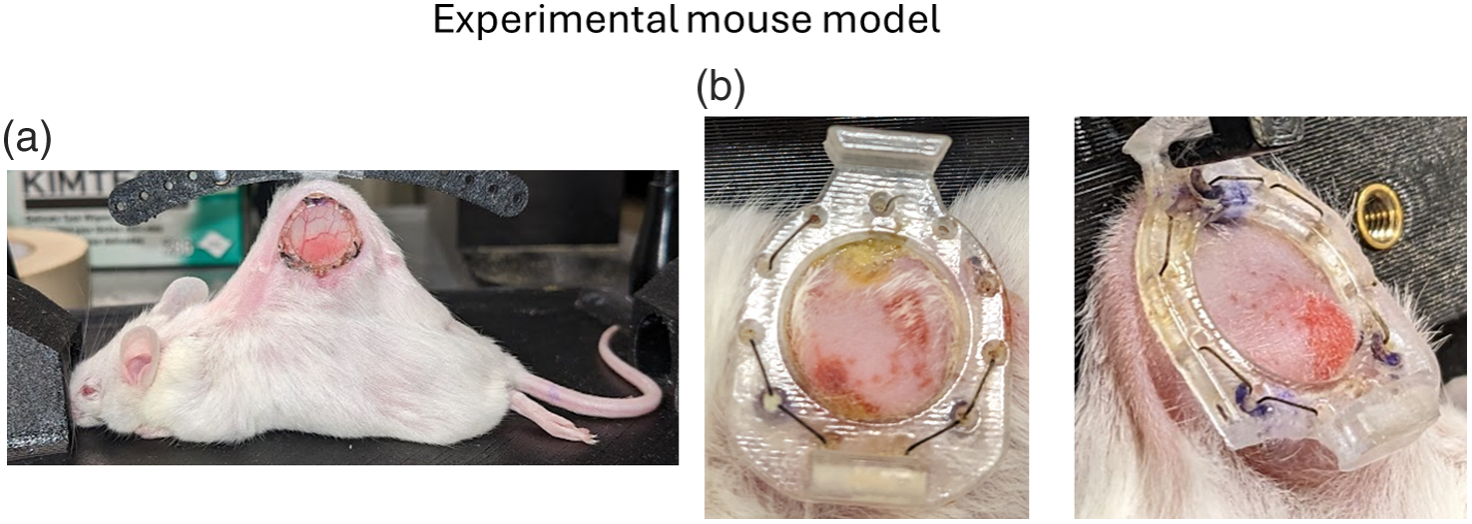
Photographs of an experimental window chamber mouse model (immunocompromised) bearing a human pancreatic ductal adenocarcinoma tumor xenograft. (a) Viewing the window side whereby the tumor is directly visible through the transparent window. (b) Viewing the back side of the window chamber, which does not contain a window, with the tumor beneath non-flat layers of skin, fur, and scar tissue; this presents more challenging imaging conditions.

All animal procedures were performed in accordance with appropriate standards under protocols approved by the University Health Network Institutional Animal Care and Use Committee in Toronto, Canada (animal use protocols #3256). Used in this study were three female mice (7 to 8 weeks old), which were immunocompromised $\mathrm{NOD}\text{-}{\mathrm{Rag}1}^{\text{null}}{\mathrm{IL}2\mathrm{rg}}^{\text{null}}$ (NRG). Each mouse contained a heterotopic pancreatic ductal adenocarcinoma (PDAC) xenograft in the dorsal skin, which were ∼2 to 3 mm thick as measured by optical coherence tomography, encapsulated by a window chamber for imaging, as shown in Fig. 1 (see Refs. 50 and 51 for details on tumor inoculation and window chamber design). Anesthesia in the mice was initiated using 5% isoflurane and maintained with 1.5% isoflurane.

### 2.3 Tumor Margin Determination

**Fig. 2:**
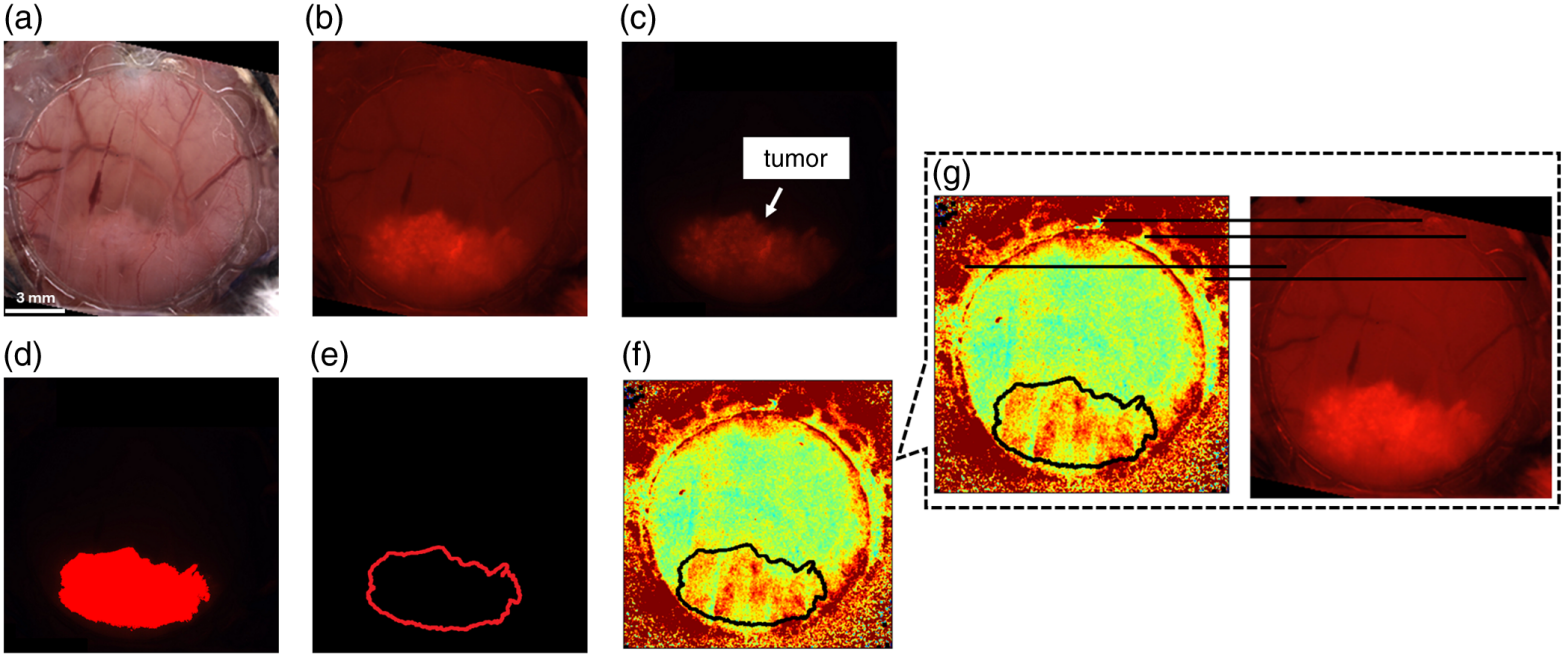
Steps performed to determine the tumor margin of each mouse, followed by co-registration of the tumor margin outline with the polarimetry images (see text for details). (a) White-light image to visualize fiducial markers (e.g., window chamber elements). (b) Image of the window chamber using white-light along with 535-nm illumination to visualize the tumor region (via fluorescence) and the fiducial markers to aid in co-registration. (c) Fluorescence-only image, depicting the tumor region. (d) Binarized image depicting the tumor region in red, generated by thresholding the fluorescence-only image. (e) Extracted tumor margin outline from the binarized image. (f) Polarimetry image (circular DOP) via 632.8-nm illumination which contains fiducial markers (e.g., window chamber elements) to enable accurate co-registration with the tumor margin outline image, as depicted in panel (g) using congruent lines (parallel and equal length lines) as guides for the eye.

To compare polarimetric signals from the tumor and non-tumor tissues, the tumor margins were determined, and their outlines were overlayed onto the polarimetric images; thus, pixels within the tumor outlines were defined as tumor tissue pixels, and the pixels outside of the tumor outlines were defined as non-tumor pixels. Figure 2 shows the sequence of images taken to determine the tumor margins; these images all correspond to mouse 1 to serve as a representative example. The tumor cell line (BxPC-3, AntiCancer Inc., San Diego, California, United States) is labeled with Ds-Red fluorescence protein which emits 580-nm light upon 535-nm excitation (see Refs. 50–52 for details). An epifluorescence microscope (Leica Microsystems MZ FLIII, Richmond Hill, Ontario, Canada) was used to obtain the brightfield and fluorescence images.

A white-light image (a) is taken as a reference which shows fiducial markers (e.g., window chamber mounting elements). To aid in image co-registration, (b) an image of the window chamber under white-light and 535-nm illumination is taken with a bandpass filter (620-nm central wavelength and 60-nm bandwidth) placed before the detector to show the fluorescence component (highlighting the tumor region) along with the window chamber elements (i.e., fiducial markers). Then, a fluorescence-only image (c) is captured using only 535-nm illumination and bandpass-filtered detection, which only shows the fluorescing tumor region. A threshold was applied to the fluorescence-only image to generate a binary image (d), displaying the tumor (red) and non-tumor (black) regions. The threshold was set such that any pixel with 95% intensity (relative to the maximum pixel intensity) was color-coded as solid red; any other pixel was set to black. Finally, using the binary image, an outline was extracted (e) whereby the red pixels on the border (5-pixel-thick) were kept, and the inner pixels were set to black. In the future, this binarized tumor/healthy image should be validated against histology to ensure the accuracy of tumor delineation.

The tumor outline images were co-registered to the polarimetric images, such as the one shown in Fig. 2(f) by aligning the fiducial markers which were present in both the white-light images (and the white-light + fluorescence images) and the polarimetry images, as shown in Fig. 2(g), for example, using the semi-circular ring-like elements on the rim of the window chambers as fiducial markers. By co-registering the white-light images to the polarimetry images, the tumor outline images could also be co-registered because they were already co-registered to the white-light images.

### 2.4 Calculations: Polarization Metrics and Receiver Operator Curves

#### 2.4.1 Polarization metrics

The circular DOP we use here ranges from −1 to +1 and is calculated as

$$\mathrm{DOCP}=\frac{S_{3,\text{in}}}{\left|S_{3,\text{in}}\right|}\cdot \frac{S_{3,\text{out}}}{S_{0,\text{out}}}=\frac{\{I_{R}-I_{L}{\}}_{\text{in}}}{\left|\{I_{R}-I_{L}{\}}_{\text{in}}\right|}\cdot \frac{\{I_{R}-I_{L}{\}}_{\text{out}}}{\{I_{R}+I_{L}{\}}_{\text{out}}},$$ (1)

where $S_{3,\text{out}}/S_{0,\text{out}}$ is the standard calculation of circular DOP; however, we multiply it by the term $S_{3,\text{in}}/|S_{3,\text{in}}|$ to assign a negative value in the case of helicity-flipping or a positive value in the case of helicity preservation.

The linear DOP also ranges from −1 to +1 and is calculated as

$$\mathrm{DOLP}=\frac{-S_{1,\text{in}}}{\left|S_{1,\text{in}}\right|}\cdot \frac{S_{1,\text{out}}}{S_{0,\text{out}}}+\frac{S_{2,\text{in}}}{\left|S_{2,\text{in}}\right|}\cdot \frac{S_{2,\text{out}}}{S_{0,\text{out}}}=\frac{-\{I_{H}-I_{V}{\}}_{\text{in}}}{\left|\{I_{H}+I_{V}{\}}_{\text{in}}\right|}\cdot \frac{\{I_{H}-I_{V}{\}}_{\text{out}}}{\{I_{H}+I_{V}{\}}_{\text{out}}}+\frac{\{I_{+45}-I_{-45}{\}}_{\text{in}}}{\left|\{I_{+45}+I_{-45}{\}}_{\mathrm{in}}\right|}\cdot \frac{\{I_{+45}-I_{-45}{\}}_{\text{out}}}{\{I_{+45}+I_{-45}{\}}_{\text{out}}},$$ (2)

where $\left(-S_{1,\text{in}}/|S_{1,\text{in}}|)\cdot (S_{1,\text{out}}/S_{0,\text{out}}\right)$ and $\left(S_{2,\text{in}}/|S_{2,\text{in}}|)\cdot (S_{2,\text{out}}/S_{0,\text{out}}\right)$ become negative when there is higher co-linear polarization intensity than cross-linear polarization intensity in the scattered light (for example, when $\{I_{V}{\}}_{\text{out}}$ is greater than $\{I_{H}{\}}_{\text{out}}$ with incident linear vertically polarized light). This enables fair comparison between Stokes linear and circular DOP because both will now take on negative values upon direct backscatter events such as specular reflection and remain positive otherwise.

Details on the linear and circular DOP calculations can be found in Refs. 6 and 40. Also, details on Mueller matrix calculations can be found in Ref. 6. In this study, the Mueller matrix is indexed from M11 to M44, where the first number indicates the row and the second number indicates the column (e.g., M34 = Mueller matrix element at row 3 and column 4).

#### 2.4.2 Receiver operator curves

The classification performance between tumor and non-tumor of circular DOP can be done at the pixel level for each circular DOP image using receiver operating characteristic (ROC) curves which plots values of (x,y) = (1−specificity, sensitivity), as follows. A circular DOP value can be chosen as a “classification threshold setting” which then enables the counting of true positive (TP), true negative (TN), false positive (FP), and false negative (FN) pixels. TP pixels are pixels located within the defined tumor region (via the fluorescence threshold) with values that are above the given circular DOP threshold setting, whereas TN pixels are considered pixels located in the non-tumor region with values below the given circular DOP threshold setting. FP pixels are pixels in the non-tumor region with values above the given circular DOP threshold, and FN pixels are pixels within the tumor region with values below the given circular DOP threshold. From the TP, TN, FP, and FN pixel counts, a specificity and sensitivity calculation can be performed as sensitivity = $\frac{\mathrm{TP}}{\mathrm{TP}+\mathrm{FN}}$ and specificity = $\frac{\mathrm{TN}}{\mathrm{TN}+\mathrm{FP}}$. The circular DOP classification threshold setting can be varied from −1 to +1 (i.e., the full range of possible circular DOP values) in steps of 0.05 (−0.95,−0.90,…,+0.90,+0.95), and for each threshold setting, a sensitivity and specificity calculation can be performed and then used to plot (x,y) = (1−specificity, sensitivity), thereby producing a ROC curve. The confidence intervals corresponding to each area under the curve (AUC) value are very tight (all below 0.006), likely due to the large sample size of pixels ($>5\times 10^{5}$); thus, we do not quote them in the Sec. 3. We refer the reader to Ref. 53 for details on this classification evaluation method for cancer/non-cancer classification applications.

#### 2.4.3 Frequency distribution histograms

Frequency distribution histograms show the distribution of pixel intensity values in the tumor (color-coded as red) and the non-tumor (color-coded as green) regions in a polarimetric image. The tumor and non-tumor histograms are each normalized such that the area under each curve equals 1. To gain insight into the tumor/healthy contrast yielded by a polarization metric, the area of overlap between each tumor and non-tumor histogram is calculated, ranging between 0 (no overlap, total separation) and 1 (total overlap, no separation). Thus, in this scheme, lower areas of overlap imply improved segmentation/separation.

## 3 Results and Discussion

**Fig. 3:**
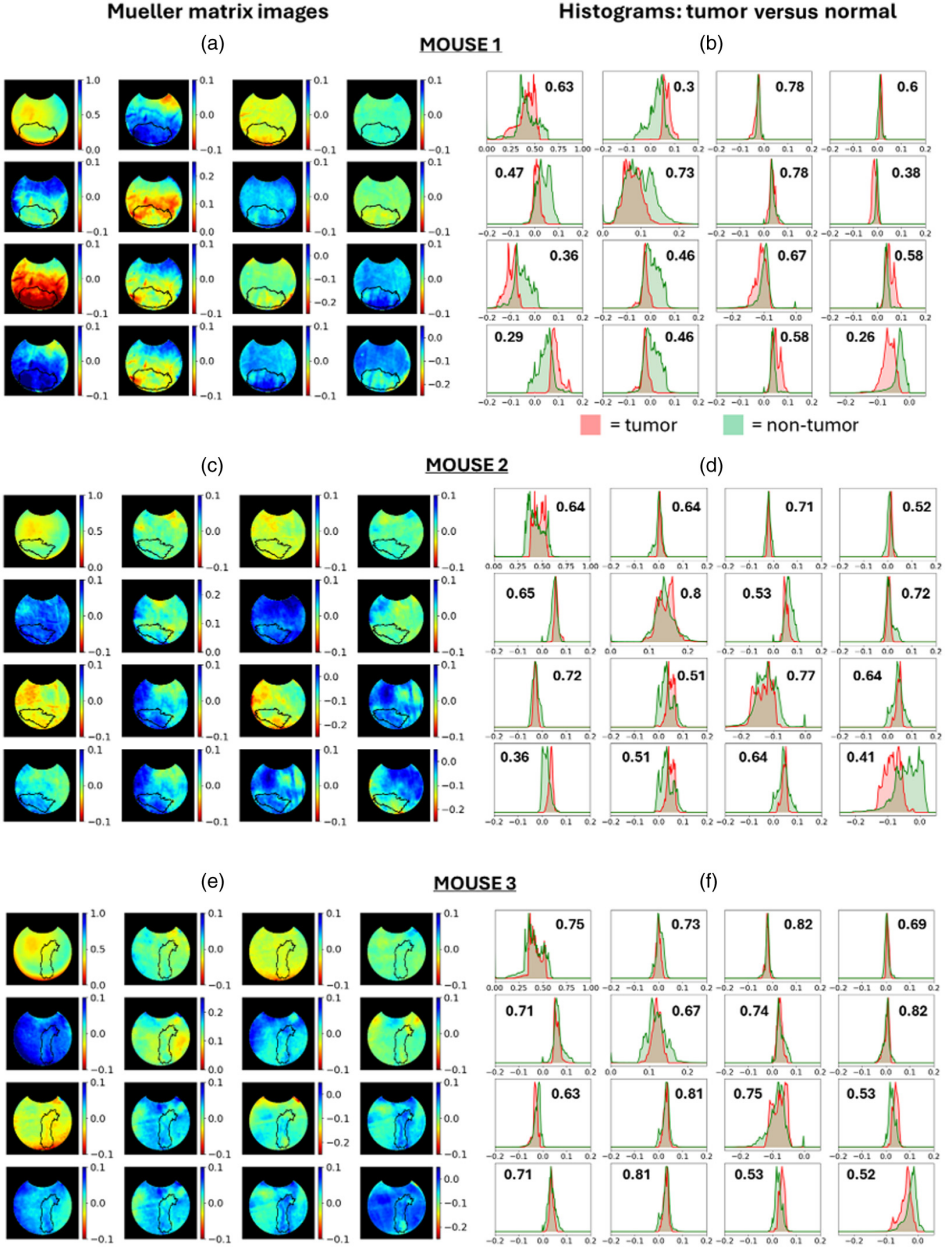
Mueller matrix images of mouse 1, 2, and 3 (a), (c), and (e) enable comparison of polarimetric elements to determine which, if any, separates tumor tissue from non-tumor tissue; the black contour depicts the tumor border. The histograms (b), (d), and (f) correspond to each Mueller matrix element. The inset values indicate the area of overlap between the tumor and non-tumor histograms; thus, the element with the least overlap yields the greatest separation. It is seen that M44, which essentially quantifies the circular DOP, yields the most consistent separation between the two tissue types.

**Fig. 4:**
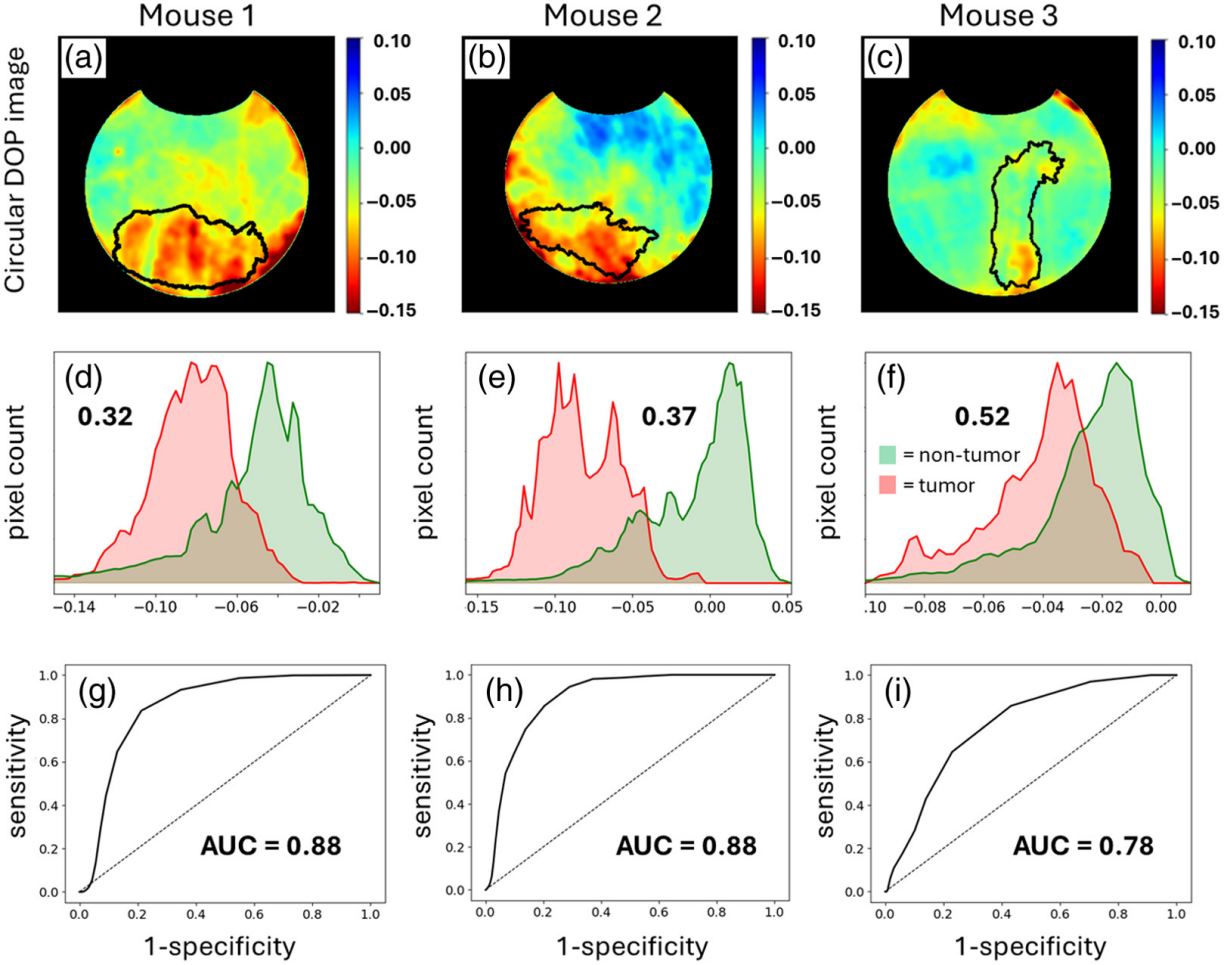
Circular DOP images of mice 1 to 3 (a)–(c) through the transparent window, whereby the tumor xenografts are directly visible. Corresponding histograms (d)–(f) indicating the numbers of pixels in the tumor (red shade) and non-tumor (green shade) regions; the inset values indicate the area of overlap between the tumor and non-tumor histograms; thus, the element with the least overlap yields the greatest separation. (g)–(i) ROC curves with associated AUC values to quantify the separation between tumor and non-tumor tissues (see Sec. 2 for details).

To compare how well the different polarimetric measurements separate tumor tissue from non-tumor tissue, we first analyze the full Mueller matrix set of images for each mouse from the front/window side, shown in Figs. 3(a), 3(c), and 3(e); the black contour indicates the tumor border. Corresponding to each Mueller matrix image are frequency distribution histograms of pixels that are within the tumor (red shade) and within the non-tumor (green shade) regions as shown in Figs. 3(b), 3(d), and 3(f); the inset text values indicate the area of overlap between the tumor and non-tumor histograms; thus, the Mueller element with the lowest value yields the greatest separation between tumor and non-tumor. Upon examination of the histograms in Figs. 3(b), 3(d), and 3(f), M44 most consistently yields the lowest area of overlap (or second-lowest for mouse 2) and thus achieves the best separation between tumor and non-tumor. M44 is highly related to circular DOP, ranging from −1 (helicity-flipped light) to +1 (helicity-preserved light);^54^^,^^55^ however, circular DOP is a simpler calculation requiring only two measurements [see Eq. (1)], as opposed to 16 measurements required to calculate the Mueller matrix. As circular DOP is simpler to calculate and is quite similar to M44—for example, as will be seen in Figs. 4(d)–4(f)—circular DOP images yield similar degrees of separation between tumor and non-tumor as the corresponding M44 images in Figs. 3(b), 3(d), and 3(f); thus, we proceed to analyze circular DOP images of each mouse.

Figure 4 shows the circular DOP images of mice 1 to 3 (a–c) along with the tumor (red shade) and non-tumor (green shade) histograms (d–f). It is evident that circular DOP does not enable direct tumor margin delineation—this remains to be a challenging feat as there is yet to be a demonstration of polarimetric tumor margin delineation in bulk tissue. However, there appears to be a noticeable contrast between the tumor and non-tumor regions in the circular DOP images. Specifically, notice in Figs. 4(d)–4(f) that the tumor histograms take on higher magnitude and more negative circular DOP values relative to the non-tumor histograms. This contrast is quantified by ROC curves, shown in Figs. 4(g)–4(i), with accompanying AUC values (see Sec. 2 for details on ROC and AUC calculations). AUC values >0.85 are generally regarded as yielding strong separation,^56^ as exhibited in mice 1 and 2, whereas acceptable separation is achieved for mouse 3 (AUC = 0.77).^56^ It is important to note that these findings are based on a limited sample size of only three mice, a factor that warrants further consideration for generalizability.

The higher magnitude circular DOP values are in agreement with the commonly observed phenomenon of higher polarization preservation in tumor tissue.^4^^,^^7^^–^^11^^,^^14^ The polarization preservation is mainly limited to circular polarization in this case; however, linear DOP is also somewhat higher in magnitude in the tumors as discussed below [see Figs. 6(c) and 6(d)]. Importantly, this polarization preservation cannot arise from higher absorption in the tumor tissue, as has been theorized,^19^^–^^24^ for example, due to deoxyhemoglobin, which can increase in tumors^57^ and readily absorbs 632.5-nm light.^58^ This is because there are no observed decreases in M11 intensity (i.e., unpolarized intensity) in those regions (see the M11 images and corresponding histograms, Fig. 3). The more negative circular DOP values in tumor tissue seen in Fig. 4 have often been observed^10^^,^^14^^,^^42^^,^^43^^,^^59^ which indicates increased helicity-flipping. As seen in Refs. 17, 27, 28, 32, 33, 60, and 61, this suggests that tumor tissues comprised smaller-sized scatterers.

The tissues in Fig. 4 were imaged under favorable conditions via the window chamber, which replaces each mouse’s skin and fur with a transparent window to enable direct optical access to the tumor xenografts. Furthermore, the tissues of interest are relatively flat due to its contact with the window, which reduces any signal artifacts arising from changes in curvature.^62^ However, such conditions are not clinically realistic, for example, in the context of surgical or procedural guidance, tumor tissues are seldom perfectly flat and completely exposed; instead, they are often irregularly shaped and lie fully or partially beneath layers of non-tumor tissue. Thus, it is important to investigate the prospect of polarimetric imaging of sub-surface tumors that lie beneath non-flat tissue layers. To do so, we image the tumor xenografts through the non-window side (i.e., back side) of the chamber, whereby the tumors lie beneath skin and fur with a non-flat surface [e.g., see Fig. 1(b)].

**Fig. 5:**
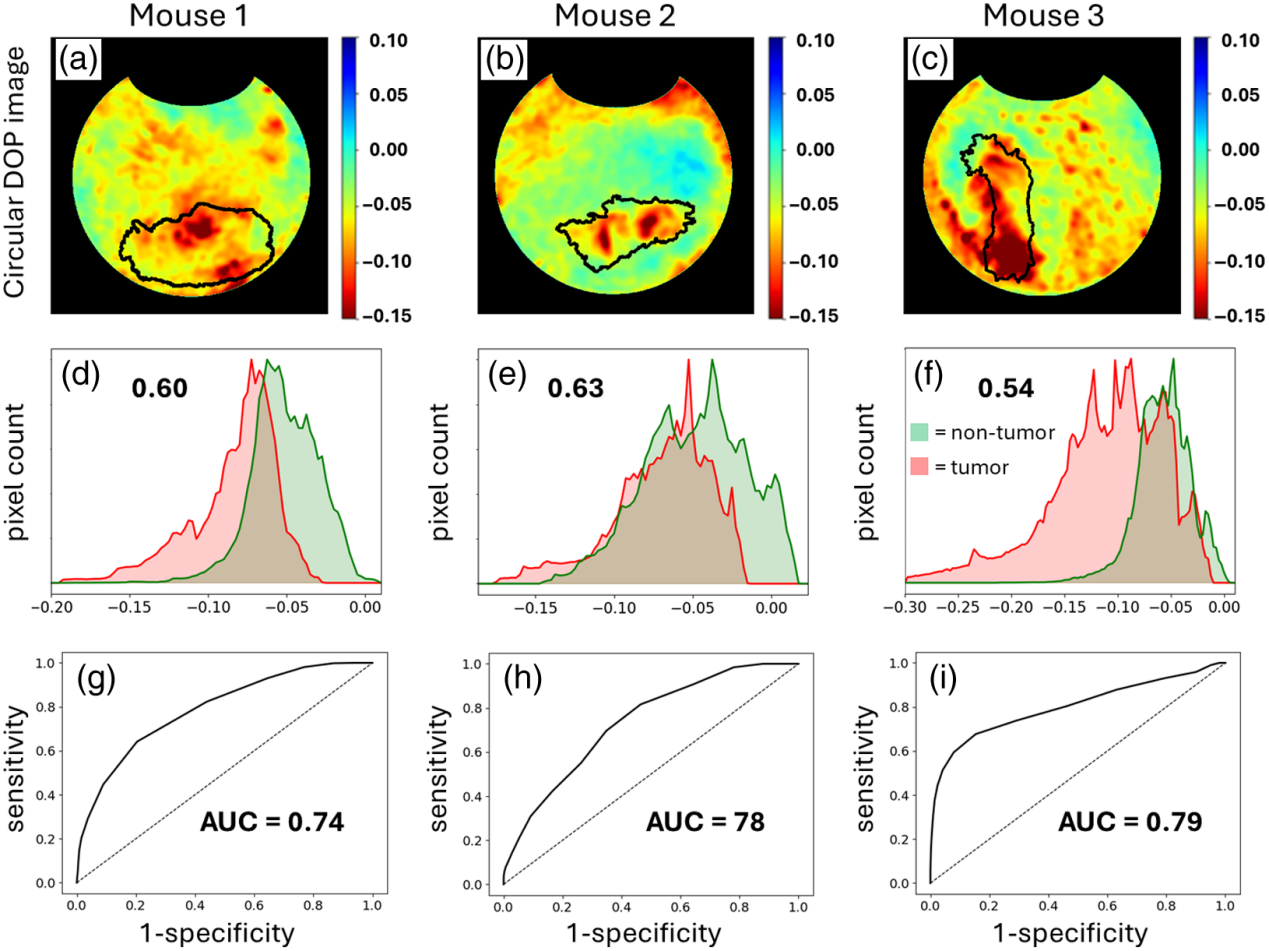
Circular DOP images (a)–(c) from the back side of the window chamber, whereby the tumor xenografts lie beneath non-flat layers of skin, fur, and scar tissue. Corresponding histograms (d)–(f) indicating the numbers of pixels in the tumor (red shade) and non-tumor (green shade) regions; the inset values indicate the area of overlap between the tumor and non-tumor histograms; thus, the element with the least overlap yields the greatest separation. ROC curves with associated AUC values (g)–(i) to quantify the separation between tumor and non-tumor tissues.

Figures 5(a)–5(c) show the circular DOP images from the back side of mice 1 to 3. Accompanying these images are the histograms of the tumor (red shade) and non-tumor (green shade) regions, shown in Figs. 5(d)–5(f). Again, it is clear that circular DOP does not directly delineate the tumor margins; however, there is contrast in the general tumor regions. It is striking that the tumor regions still generate modest contrast, despite being covered by non-flat layers of skin, fur, and scar tissue. This contrast is seen in the histograms (d–f) and quantified by the ROC curves and accompanying AUC values which are all above 0.73, indicating useful separation.^56^ Again, it is seen that circular DOP takes on higher magnitude negative values in tumor tissue, indicating higher polarization preservation and suggesting that tumors comprised smaller-sized scatterers (due to helicity flipping). There is thus potential in utilizing polarimetric imaging to identify suspicious lesions beyond the surface of an irregularly shaped tissue—this, for example, may be useful in guiding more specific, but slow, diagnostic tools to regions of interest.^6^

**Fig. 6:**
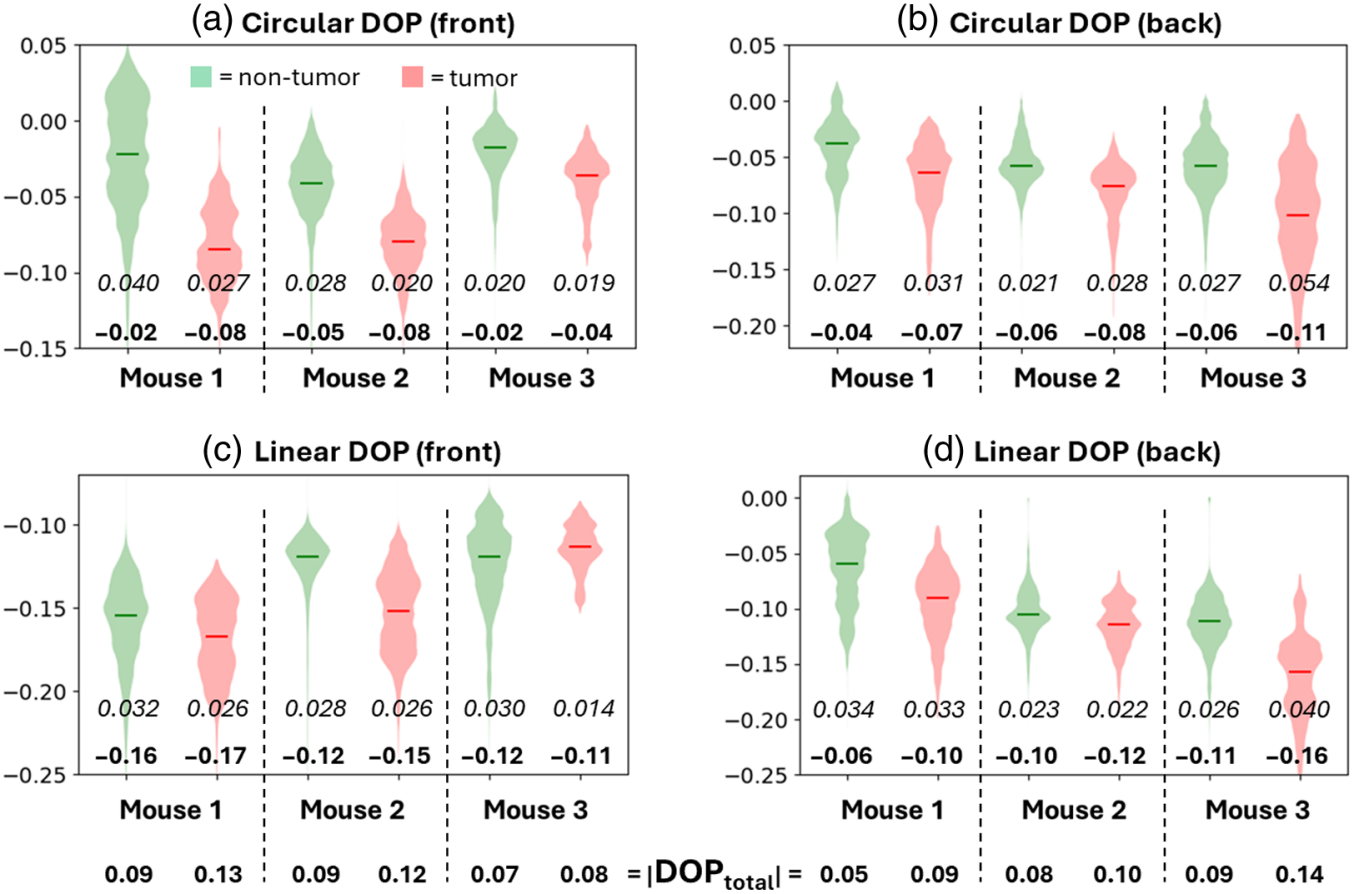
Violin plots containing circular DOP and linear DOP pixel values from the tumor (red shade) and non-tumor (green shade) regions of each mouse, imaged through the window [(a) circular DOP and (c) linear DOP] and from the back side [(b) circular DOP and (d) linear DOP]. The bolded inset text values indicate the mean values of each distribution, and the italicized inset text values indicate the standard deviation of each distribution. The bottom-most row of values indicates the total DOP values in magnitude, |DOP_total_|.

Figure 6 shows violin plots of the pixels from the tumor (red shade) and the non-tumor (green shade) regions of the circular (a and b) and linear (c and d) polarization images of the front and back sides of each mouse; the bolded inset text values indicate the mean of each violin plot and the italicized inset text values indicate the standard deviations, and the bottom-most row of bolded text values are the total DOP values in magnitude, $\left|{\mathrm{DOP}}_{\text{total}}\right|$, calculated as the average of |linear DOP| and |circular DOP|. Note that the y-axis ranges of DOP values are different in each panel for optimal visualization.

As observed in many prior studies,^4^^,^^7^^–^^11^^,^^13^^–^^15^ the DOPs from each tumor tissue are higher than the DOPs from each non-tumor tissue. Also, expectedly from the results above (Figs. 4 and 5), we can see that circular polarization offers greater separation between tumor and non-tumor regions than does linear polarization; this has been seen in Refs. 14 and 59. The AUC values for the linear DOP images are mouse 1: AUC = 0.82 (front), AUC = 0.64 (back); mouse 2: AUC = 0.62 (front), AUC = 0.74 (back); and mouse 3: AUC = 0.40 (front), AUC = 0.86 (back). In comparison, the AUC values for the circular DOP images are all higher except in the case of the back of mouse 3 (see Figs. 4 and 5). Also, notice that the circular DOP distribution corresponding to each tumor is more negative but higher in magnitude than that of each non-tumor counterpart, whereas linear DOP is only slightly higher in magnitude and more negative for each tumor relative to each non-tumor counterpart (except for mouse 3, front).

Thus, we find several indications of enhanced Rayleigh scattering in the tumor tissues, as follows. (1) There is more relative helicity flipping in tumor regions (indicated by the more negative circular DOP), which is more prevalent for media of small Rayleigh-like scatterers, as shown in previous studies.^17^^,^^27^^,^^28^^,^^32^^,^^33^^,^^60^^,^^61^ (2) Linear polarization preservation is higher in magnitude than circular polarization preservation for tumor regions, as previously observed;^10^^,^^41^ note that this is also the case in non-tumor regions, but less so. As seen in previous studies,^17^^,^^27^^,^^63^^–^^66^ this is typical of Rayleigh scattering media. (3) Co-linear intensity is higher than cross-linear intensity (indicated by the more negative linear DOP values; see Eq. (2) and related text in Ref. 40 for details) in tumor regions (except for mouse 3, front), which occurs in media of smaller Rayleigh-like scatterers, as observed in previous studies.^27^^,^^30^^,^^63^^,^^67^^,^^68^ In Ref. 40, it was observed that overall DOP is higher in Rayleigh scattering media than in larger scattering media; thus, if indeed tumor tissue comprised Rayleigh-like scatterers as the depolarization signatures suggest, then Rayleigh-like scattering may be a major contributor to the consistently observed phenomenon of higher polarization preservation in tumor tissue.^4^^,^^7^^–^^11^ In addition, circular DOP may yield greater contrast between tumor and non-tumor tissue than linear DOP due to its well-observed sensitivity to scatterer size.^27^^–^^39^^,^^69^

## 4 Conclusion

Herein, it is shown that circular DOP images yield useful contrast between PDAC tumor xenografts and surrounding healthy skin in live mice. This was observed by imaging through a window chamber to optimize imaging conditions by enabling direct optical access to each tumor. To further assess circular DOP tumor localization performance under more challenging conditions, the tumors beneath the irregularly shaped skin, fur, and scar tissue of the mice (i.e., the back, non-window side of the chamber) were also imaged; although exhibiting lower contrast, but reasonable separation was still evident. Each tumor region exhibited higher magnitude circular DOP (and total DOP) values, as has been observed in multiple reflection-mode polarimetry studies. We noted three indications of Rayleigh scattering in the tumor tissue: (1) increased helicity-flipping relative to helicity-preservation intensity, (2) higher linear DOP than circular DOP, and (3) higher co-linear intensity than cross-linear intensity. Rayleigh scatterers have been found to be highly polarization preserving; thus, we posit that higher DOP in tumor tissues may arise from an increased presence of Rayleigh scatterers. Overall, this study serves as a stepping stone toward understanding depolarization-based contrast between tumor and non-tumor tissues.

A limitation of the current pilot study investigation is the small sample size of only three mice. Although the experimental setup was rigorously designed and the observed trends are consistent across the tested animals and align with previous research on depolarization in malignant tissues, the limited number of animals may impact the statistical robustness and generalizability of the findings. Future investigations should aim to incorporate a larger sample size to enhance the statistical strength and confirm the reproducibility of these early study results.

## Data Availability

The datasets used for this study are not publicly available at this time but may be obtained upon reasonable request.
